# Cellular prion protein in human plasma–derived extracellular vesicles promotes neurite outgrowth *via* the NMDA receptor–LRP1 receptor system

**DOI:** 10.1016/j.jbc.2022.101642

**Published:** 2022-01-25

**Authors:** Steven L. Gonias, Michael A. Banki, Pardis Azmoon, Haylie K. Romero, Christina J. Sigurdson, Elisabetta Mantuano, Wendy M. Campana

**Affiliations:** 1 Department of Pathology, University of California San Diego, La Jolla, California, USA; 2 Department of Anesthesiology and Program in Neurosciences, University of California San Diego, La Jolla, California, USA; 3 San Diego Veterans Administration Healthcare System, San Diego, California, USA

**Keywords:** low-density lipoprotein receptor–related protein-1, NMDA receptor, cellular prion protein, extracellular vesicles, exosome, signal transduction, neurite outgrowth, α2M, α2-macroglobulin, ERK1/2, extracellular signal–regulated kinase 1/2, EV, extracellular vesicle, FBS, fetal bovine serum, FFP, fresh-frozen human plasma, IB, immunoblot, LRP1, lipoprotein receptor–related protein-1, NMDA, *N*-methyl-d-aspartate, NMDA-R, *N*-methyl-d-aspartate receptor, NGF-β, nerve growth factor beta, NTA, nanoparticle tracking analysis, NTC, nontargeting control, P-AC, phosphatidylserine affinity chromatography, PrP^C^, cellular prion protein, PS, phosphatidylserine, RAP, receptor-associated protein, S-PrP, recombinant soluble cellular prion protein, SEC, size-exclusion chromatography, SFM, serum-free medium, TEM, transmission electron microscopy, tPA, tissue-type plasminogen activator, UC, ultracentrifugation, UCSD, University of California San Diego

## Abstract

Exosomes and other extracellular vesicles (EVs) participate in cell–cell communication. Herein, we isolated EVs from human plasma and demonstrated that these EVs activate cell signaling and promote neurite outgrowth in PC-12 cells. Analysis of human plasma EVs purified by sequential ultracentrifugation using tandem mass spectrometry indicated the presence of multiple plasma proteins, including α_2_-macroglobulin, which is reported to regulate PC-12 cell physiology. We therefore further purified EVs by molecular exclusion or phosphatidylserine affinity chromatography, which reduced plasma protein contamination. EVs subjected to these additional purification methods exhibited unchanged activity in PC-12 cells, even though α_2_-macroglobulin was reduced to undetectable levels. Nonpathogenic cellular prion protein (PrP^C^) was carried by human plasma EVs and essential for the effects of EVs on PC-12 cells, as EV-induced cell signaling and neurite outgrowth were blocked by the PrP^C^-specific antibody, POM2. In addition, inhibitors of the *N*-methyl-d-aspartate (NMDA) receptor (NMDA-R) and low-density lipoprotein receptor–related protein-1 (LRP1) blocked the effects of plasma EVs on PC-12 cells, as did silencing of *Lrp1* or the gene encoding the GluN1 NMDA-R subunit (*Grin1*). These results implicate the NMDA-R–LRP1 complex as the receptor system responsible for mediating the effects of EV-associated PrP^C^. Finally, EVs harvested from rat astrocytes carried PrP^C^ and replicated the effects of human plasma EVs on PC-12 cell signaling. We conclude that interaction of EV-associated PrP^C^ with the NMDA-R–LRP1 complex in target cells represents a novel mechanism by which EVs may participate in intercellular communication in the nervous system.

Extracellular vesicles (EVs) are produced by diverse cells and include exosomes, which form by inward budding of multivesicular bodies in the endosomal transport pathway, microvesicles that shed from the cell surface, and membrane blebs formed by apoptotic cells (1, 2). EVs have been strongly implicated in cell–cell communication, based mainly on their ability to transfer cargo from one cell to another, including mRNAs, microRNAs, and proteins (1, 2, 3, 4). Other mechanisms of EV activity have been described. For example, integrins in EV membranes not only target EVs to specific cell types but also trigger cell signaling in target cells (5). Neutrophil elastase associates with EV surfaces and efficiently degrades extracellular matrix proteins (6). Another example involves EV-associated tumor necrosis factor receptor-1, which functions similarly to soluble cytokine receptors, binding soluble tumor necrosis factor alpha and preventing it from engaging cellular receptors (7, 8). Understanding the complete spectrum of mechanisms by which EVs regulate cell physiology is an important goal.

Cellular prion protein (PrP^C^) is a widely expressed glycosylphosphatidylinositol-anchored membrane protein which, when misfolded, causes transmissible neurodegenerative diseases (9, 10, 11). PrP^C^ is carried by exosomes and other EVs produced by diverse cells and platelets (12, 13, 14, 15). The abundance of PrP^C^ in EVs may be regulated by the overall expression level in the EV-generating cell (16). PrP^C^ has been identified in EVs isolated from human blood bank plasma and the cerebrospinal fluid of sheep (17, 18, 19).

We previously showed that a recombinant protein (S-PrP), corresponding closely in sequence to a form of PrP^C^ released from cell surfaces by a disintegrin and metalloproteinase domain–containing protein 10 (20), activates cell signaling and promotes neurite outgrowth in PC-12 and N2a cells by engaging a cell-signaling receptor assembly that includes low-density lipoprotein receptor–related protein-1 (LRP1) and the *N*-methyl-d-aspartate (NMDA) receptor (NMDA-R) (21). LRP1 and the NMDA-R are well characterized as cell-signaling receptors for various soluble proteins, including tissue-type plasminogen activator (tPA), the activated conformation of α_2_-macroglobulin (α_2_M), and matrix metalloprotease-9 (22, 23, 24). LRP1 also has been reported to function in phagocytosis of large particles (25) and efferocytosis (26, 27). In this study, we demonstrate that membrane-anchored PrP^C^ in human plasma EVs engages the NMDA-R–LRP1 receptor complex in PC-12 cells to activate cell signaling and promote neurite outgrowth, similarly to S-PrP (21).

The ability of EVs to induce neurite outgrowth is previously described (28, 29, 30, 31, 32). The mechanism identified here, in which the NMDA-R–LRP1 receptor system in target cells plays an essential role, is novel. The effects of human plasma EVs on PC-12 cell signaling and neurite outgrowth were blocked by the PrP^C^-specific antibody, POM2. EVs isolated from cultured rat astrocytes also expressed PrP^C^ and activated cell signaling in PC-12 cells by a pathway that was inhibited by POM2. The interaction of EV-associated PrP^C^ with target cell NMDA-R–LRP1 receptor complex may contribute to the ability of EVs to mediate cell–cell communication in the nervous system.

## Results

### Identification of PrP^C^ in human plasma EVs

**Figure 1:**
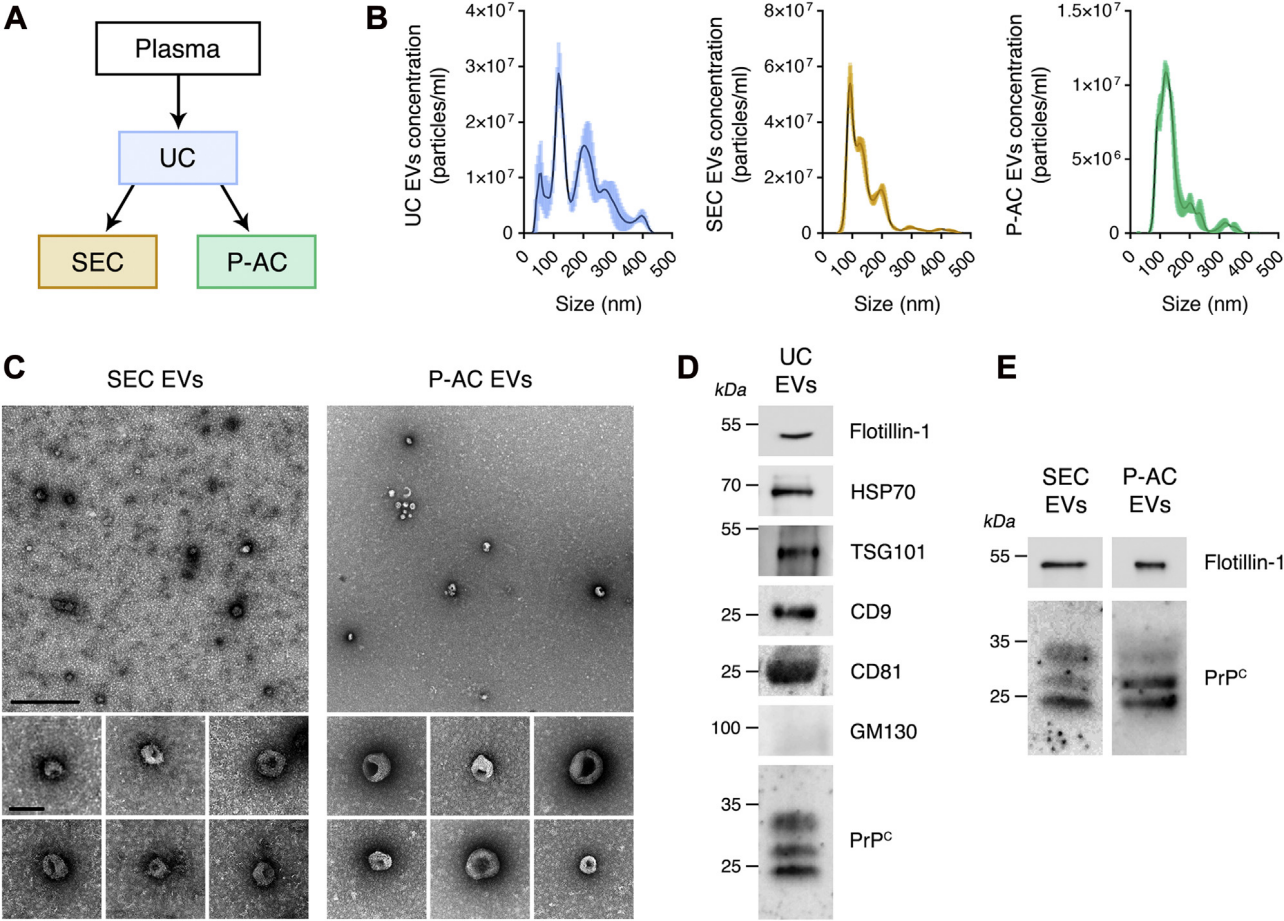
**Comparison of methods for isolating EVs from human plasma.***A*, EVs were harvested from plasma by sequential UC. For some studies, UC EVs were further purified by molecular exclusion chromatography on Sepharose CL-6B (SEC) or by immunoaffinity chromatography on PS-specific antibody, covalently coupled to Sepharose (P-AC). *B*, representative NTA studies comparing the relative abundance of EVs of various sizes in UC, SEC, and P-AC EV preparations. *C*, TEM images of SEC and P-AC EV preparations. Specimens were negatively stained with uranyl acetate (the scale bar represents 500 nm for *top images* and 100 nm for *bottom images*). *D*, immunoblot analysis of UC EVs shows the EV biomarkers, flotillin-1, heat shock protein-70 (HSP70), TSG101, CD9, and CD81. GM130 was not present, as anticipated. PrP^C^ was detected in UC EVs by immunoblot analysis following immunoprecipitation (IP/IB). *E*, immunoblot analysis showing that SEC EVs and P-AC EVs retain PrP^C^. SEC EVs were probed for PrP^C^ following IP/IB. Blots were probed for flotillin-1 to confirm the presence of EVs and as a control for EV protein load. EV, extracellular vesicle; GM130, golgi matrix protein 130; IB, immunoblot; NTA, nanoparticle tracking analysis; P-AC, phosphatidylserine affinity chromatography; PrP^C^, cellular prion protein; PS, phosphatidylserine; SEC, size-exclusion chromatography; TEM, transmission electron microscopy; TSG101, tumor susceptibility gene 101; UC, ultracentrifugation.

EVs were harvested from fresh-frozen human plasma (FFP) obtained from the University of California San Diego (UCSD) transfusion service. Initially, EVs were isolated by sequential ultracentrifugation (UC), applying minor modifications to established methods (33, 34). To further purify EVs, samples isolated by UC were subjected to molecular exclusion chromatography on Sepharose CL-6B, which has a fractionation range of 1 × 10^4^–4 × 10^6^ for globular proteins. The resulting preparations are referred to as size-exclusion chromatography (SEC) EVs. We also took advantage of the fact that the outer membranes of many EVs are rich in phosphatidylserine (PS) (35, 36, 37) and covalently coupled PS-specific antibody to Sepharose CL-4B. UC EVs that were further purified by PS immunoaffinity chromatography using immobilized PS-specific antibody are referred to as phosphatidylserine affinity chromatography (P-AC) EVs (Fig. 1*A*).

We incorporated an intermediate step into our EV harvesting method in which plasma was subjected to UC at 20,000*g*. This step was designed to selectively pellet larger EVs and enrich UC EV preparations in smaller EVs, which include exosomes; however, nanoparticle tracking analysis (NTA) demonstrated that there was still considerable size heterogeneity in the UC EVs (Fig. 1*B*). This heterogeneity was decreased in SEC EVs and P-AC EVs.

UC, SEC, and P-AC EVs were analyzed by transmission electron microscopy (TEM) after negative staining with uranyl acetate. Representative images of SEC EVs and P-AC EVs are shown in Figure 1*C*. Multiple particles with cup-like morphology were observed in all three preparations, consistent with the known ultrastructure of EVs. The granularity of the background was somewhat decreased in P-AC EV preparations, compared with UC or SEC EV preparations. This result was interpreted to reflect a lower level of plasma protein contamination in the P-AC EVs.

Immunoblot (IB) analysis of UC EVs, isolated from human plasma, demonstrated flotillin-1, which is a lipid raft–associated protein, heat shock protein-70, tumor susceptibility gene 101, and the tetraspanins, CD9 and CD81 (Fig. 1*D*). These proteins are considered EV biomarkers (38). The golgi matrix protein 130 was absent from human plasma UC EVs, as anticipated.

PrP^C^ was detected in human plasma UC EVs by IB analysis. When EV-associated PrP^C^ was immunoprecipitated using monoclonal antibodies POM2 and POM19 coupled to Dynabeads Protein-G, prior to IB analysis (immunoprecipitation/IB), up to three PrP^C^ bands were detected between 25 and 37 kDa, consistent with the known glycosylation states of PrP^C^ (39). An uncropped IB showing PrP^C^ in a UC EV preparation isolated from a different plasma sample is shown in Fig. S1. The relative abundance of the three PrP^C^ bands varied in UC EVs isolated from different plasma samples, as is evident by comparing the images in Figure 1*D* and Fig. S1. Figure 1*E* shows that PrP^C^ was retained when UC EVs were further purified to generate SEC EVs and P-AC EVs.

### Analysis of plasma protein contaminants in human plasma EV preparations

**Figure 2:**
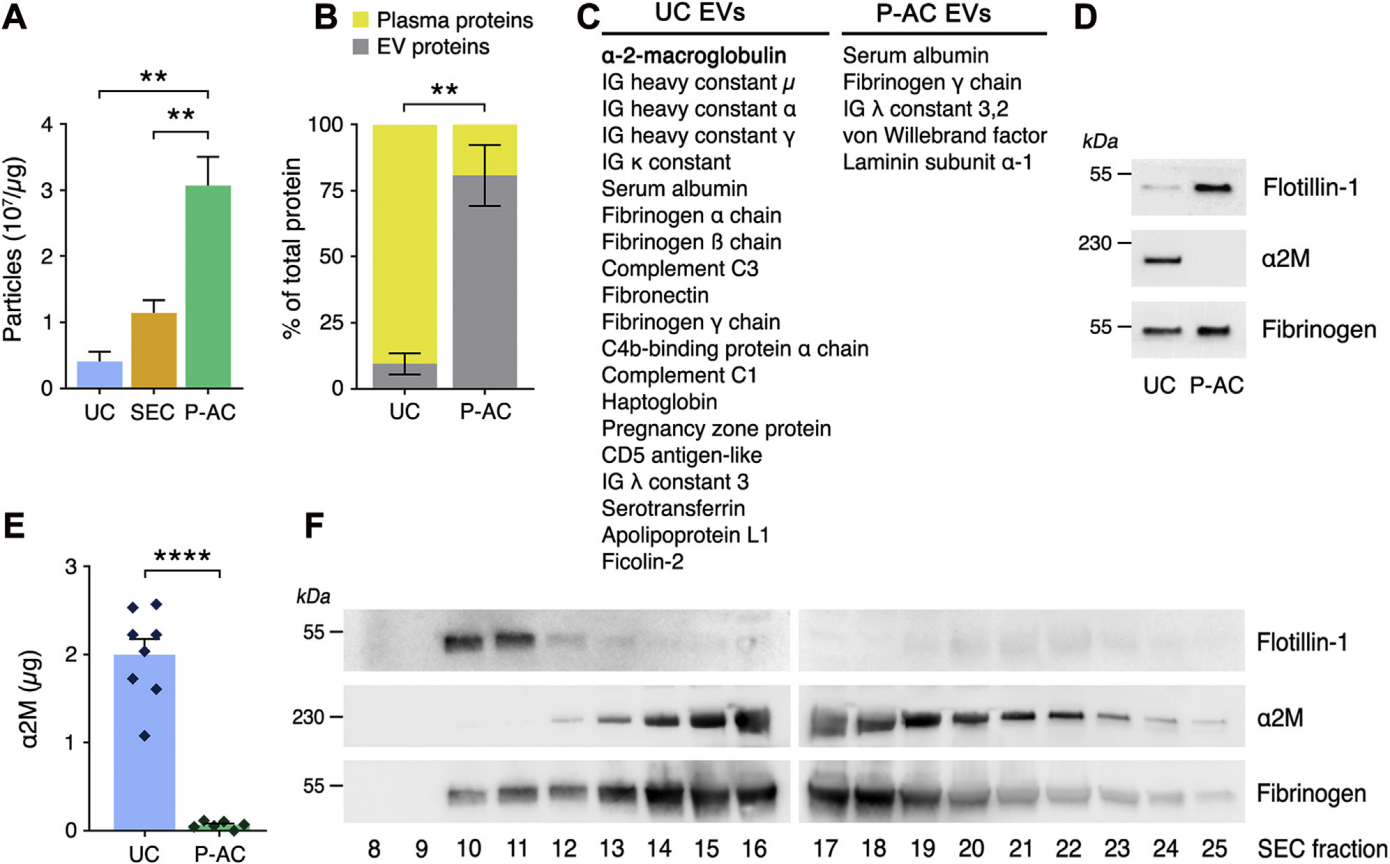
**The plasma NMDA-R–LRP1 ligand, α**_**2**_**M, is separated from plasma EVs by PS immunoaffinity chromatography.***A*, UC, SEC, and P-AC EV preparations were analyzed by NTA and BCA assay. The number of particles (assumed to be EVs) per microgram of protein was determined as an index of contamination of the EV preparations by plasma proteins (mean ± SEM, n = 3; ∗∗*p* < 0.01). *B*, UC and P-AC EV preparations were trypsinized. Tryptic peptides were identified by LC–MS/MS, and the abundance of proteins was estimated by spectral counts. Cellular proteins were assumed to be EV associated. The fraction of total protein attributable to EVs, as opposed to plasma proteins, is shown (mean ± SEM, n = 3 for UC EVs and n = 4 for P-AC EVs, ∗∗*p* < 0.01). *C*, individual proteins in EV preparations identified by LC–MS/MS were ranked according to abundance based on spectral counts. Plasma proteins that were amongst the 25 most abundant proteins in UC and P-AC EVs are listed, beginning with the most abundant (α_2_M in UC EVs). Further information relevant to *C* is presented in File S1. *D*, immunoblot analysis was performed to detect flotillin-1, α_2_M, and fibrinogen γ-chain in UC and P-AC EVs. *E*, the abundance of α_2_M in different UC and P-AC EV preparations was determined by trypsin-binding assay, in comparison to a standard curve generated with different known amounts of purified α_2_M. α_2_M levels were standardized based on EV protein content (n = 6–8; ∗∗∗∗*p* < 0.0001). *F*, UC EVs were subjected to SEC on Sepharose CL-6B. Elution fractions were examined by immunoblot analysis to detect flotillin-1, α_2_M, and fibrinogen γ-chain. α2M, α_2_-macroglobulin; BCA, bicinchoninic acid; EV, extracellular vesicle; LRP1, lipoprotein receptor–related protein-1; NMDA-R, *N*-methyl-d-aspartate receptor; NTA, nanoparticle tracking analysis; P-AC, phosphatidylserine affinity chromatography; PS, phosphatidylserine; SEC, size-exclusion chromatography; UC, ultracentrifugation.

NTA and bicinchoninic acid protein assays were performed to compare UC, SEC, and P-AC EVs from human plasma. The number of particles per microgram of EV protein was increased in SEC EVs, compared with UC EVs, and significantly increased in P-AC EVs, compared with UC or SEC EVs (Fig. 2*A*). This result was interpreted to indicate that plasma protein contamination in EV preparations is decreased by applying secondary purification methods after UC.

To identify plasma proteins present in EV preparations and quantitate these proteins compared with EV proteins, we performed LC–MS/MS studies. Cellular proteins identified by LC–MS/MS were assumed to be true EV components. Tryptic peptides derived from plasma proteins indicated either contaminants or proteins that associate with circulating EVs. The abundance of proteins identified by LC–MS/MS was estimated based on spectral counts. Figure 2*B* shows that in UC EVs, 90 ± 4% of the total protein content was attributed to plasma proteins. In P-AC EVs, plasma proteins accounted for only 19 ± 12% of the total protein identified by LC–MS/MS.

Plasma proteins that were amongst the 25 most abundant proteins in each EV preparation are shown in Figure 2*C* (additional relevant data regarding identified proteins are presented in File S1). α_2_M was the most abundant plasma protein in UC EV preparations; however, α_2_M was present only in trace quantities or not detected at all in P-AC EVs. This result is notable because although the majority of the α_2_M in plasma is in the native conformation, which does not interact with the NMDA-R–LRP1 receptor system (24, 40), when α_2_M reacts with proteases, it is recognized by LRP1 and thereby activates cell signaling and promotes neurite outgrowth in neurons and neuron-like cells (24, 41, 42, 43).

The results of our LC–MS/MS studies were confirmed in validation experiments. When equivalent amounts of protein from UC EVs and P-AC EVs were compared by immunoblotting, α_2_M was abundant in the UC EVs but undetectable in the P-AC EVs (Fig. 2*D*). By contrast, fibrinogen γ chain was detected in equal abundance in both preparations. Similar results were obtained when we probed for α_2_M in UC EVs and P-AC EVs using an activity assay that detects α_2_M based on its ability to bind trypsin (44). α_2_M was readily detected in UC EVs and present at only trace levels in P-AC EVs (Fig. 2*E*).

Next, we subjected UC EVs to molecular exclusion chromatography on Sepharose CL-6B. Elution fractions were analyzed by IB analysis. Flotillin-1, which was monitored to identify fractions that contain EVs, was detected in early elution fractions, as anticipated (Fig. 2*F*). Fibrinogen partially coeluted with the EVs, whereas α_2_M eluted in later fractions even though fibrinogen and α_2_M have similar hydrodynamic radii (45, 46). This result suggests that a portion of the fibrinogen detected in plasma EV preparations was probably EV associated, whereas α_2_M appears to be mostly a contaminant.

### Human plasma EVs promote PC12 cell neurite outgrowth by engaging the NMDA-R–LRP1 receptor system

**Figure 3:**
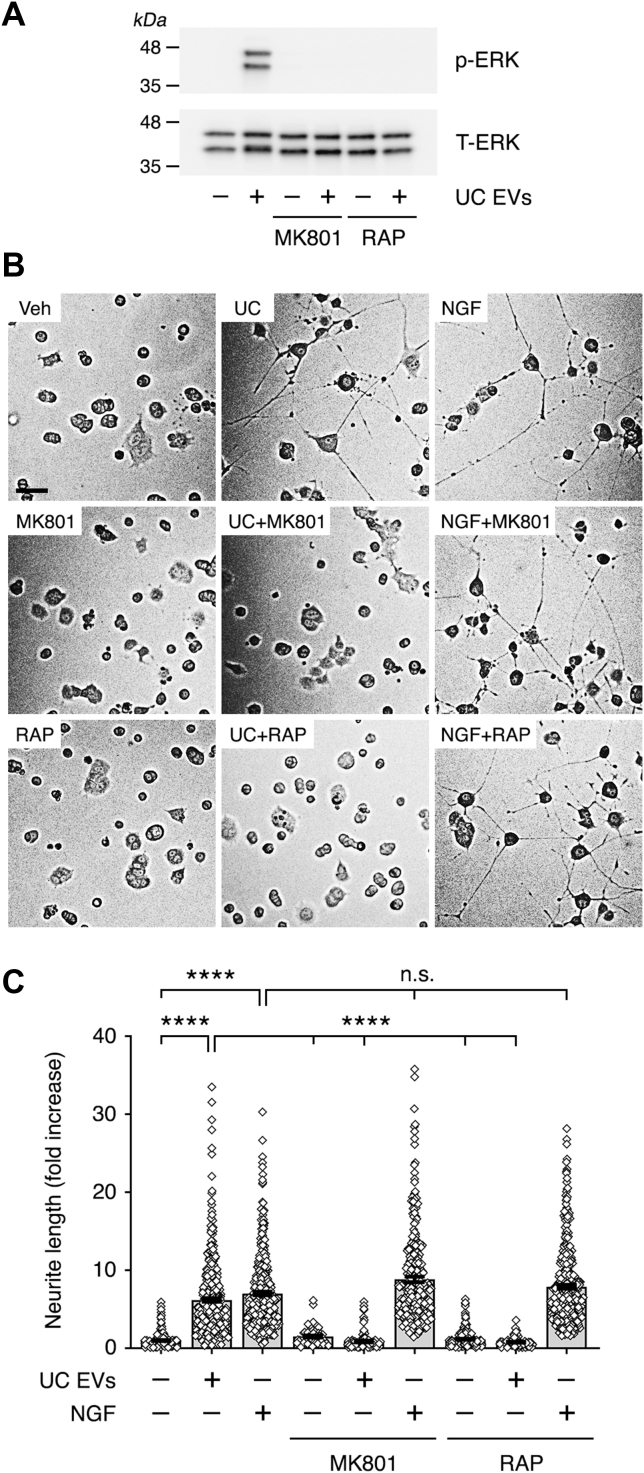
**Human plasma UC EVs activate ERK1/2 and promote neurite outgrowth in PC-12 cells by a mechanism that requires the NMDA-R–LRP1 complex.***A*, PC-12 cells were pretreated with MK801 (1.0 μM) or with RAP (150 nM) for 30 min, as indicated, and then with UC EVs (2.5 μg/ml) for 30 min. Phospho-ERK1/2 and total ERK1/2 were determined by immunoblot analysis. *B*, PC-12 cells were treated with UC EVs (2.5 μg/ml) or NGF-β (50 ng/ml) in the presence and absence of RAP (150 nM) or MK801 (1.0 μM), as indicated, for 48 h. Neurite outgrowth was examined. Representative images of cells are shown (the scale bar represents 50 μm). *C*, neurite outgrowth was quantified. For each replicate, neurites in 100 randomly selected cells were measured (data are expressed as “fold increase” compared with untreated control cells; mean ± SEM; n = 3; ∗∗∗∗*p* < 0.0001; ns, not significant). ERK1/2, extracellular signal–regulated kinase 1/2; EV, extracellular vesicle; LRP1, lipoprotein receptor–related protein-1; NGF-β, nerve growth factor beta; NMDA-R, *N*-methyl-d-aspartate receptor; RAP, receptor-associated protein; UC, ultracentrifugation.

S-PrP activates cell signaling and promotes neurite outgrowth in PC-12 cells (21). Because we and others (17, 18) identified PrP^C^ in human plasma EVs, we undertook experiments to test whether EVs replicate the activity of S-PrP. To begin, we studied EVs purified by UC alone. When PC-12 cells were treated with UC EVs (2.5 μg/ml) for 0.5 h, extracellular signal–regulated kinase 1/2 (ERK1/2) was activated (Fig. 3*A*). The response was blocked by the noncompetitive NMDA-R inhibitor, MK801/dizocilpine, and by the LRP1 inhibitor, receptor-associated protein (RAP) (23, 47).

UC EVs (2.5 μg/ml) promoted PC-12 cell neurite outgrowth, as shown in representative images in Figure 3*B* and in summary form in Figure 3*C*. RAP and MK801 entirely blocked the effects of UC EVs on neurite outgrowth. As a positive control, we also examined nerve growth factor beta (NGF-β) (50 ng/ml). NGF-β promoted neurite outgrowth as anticipated, and the response was not inhibited by RAP or MK801.

**Figure 4:**
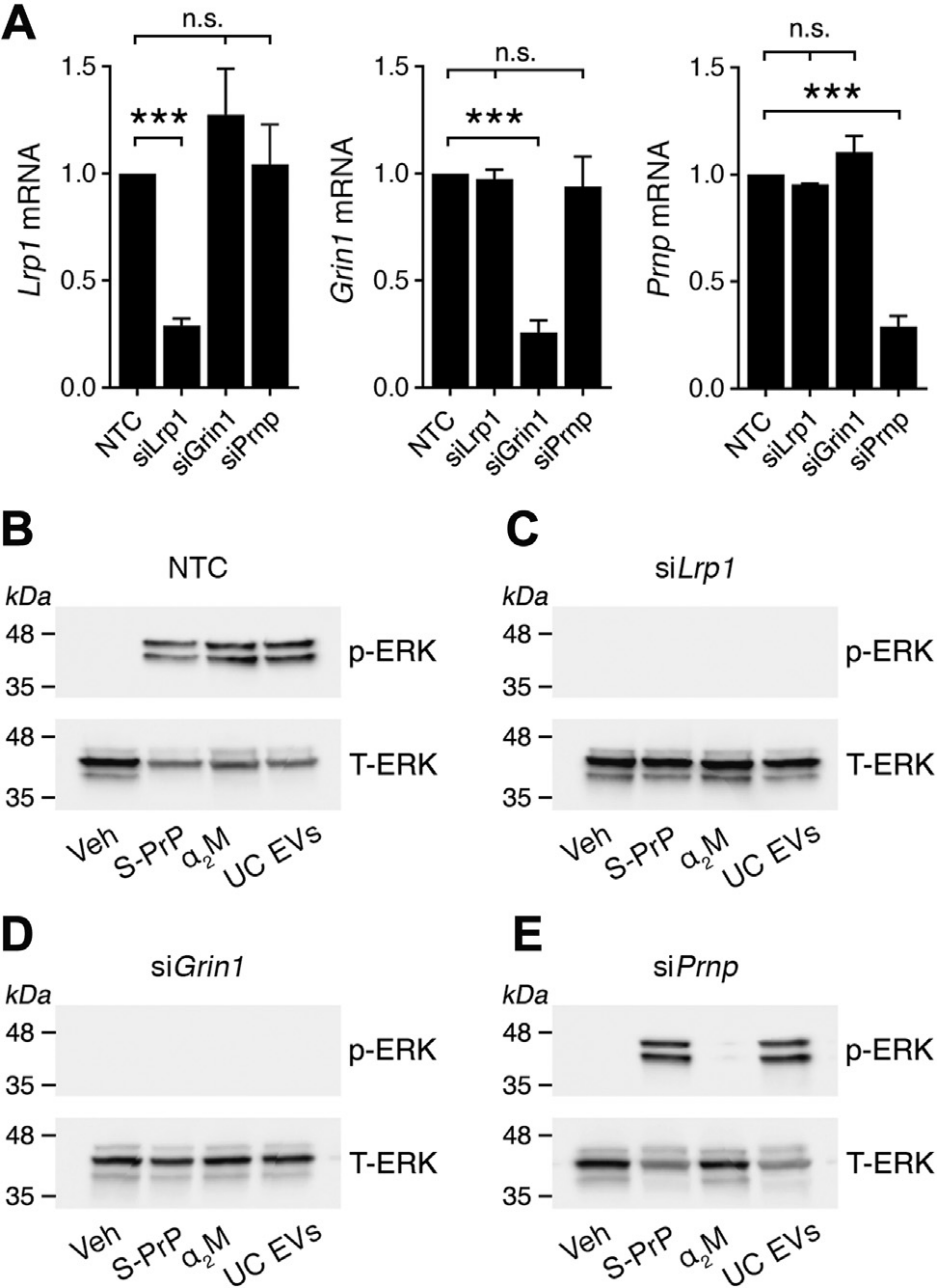
**Silencing expression of the genes encoding LRP1 or the essential GluN1 NMDA-R subunit in PC-12 cells blocks ERK1/2 activation by UC EVs.***A*, PC-12 cells were transfected with siRNA specifically targeting *Lrp1*, *Grin1*, or *Prnp*. Control cells were transfected with NTC siRNA. Expression of the mRNAs encoding LRP1, the GluN1 NMDA-R subunit, and PrP^C^ was determined 48 h later by RT–quantitative PCR (mean ± SEM, n = 3, ∗∗∗*p* < 0.001). *B*–*E*, PC-12 cells were transfected with NTC siRNA or with the targeting siRNA shown above each immunoblot. The concentration of NTC siRNA in panel *A* was 300 nM (equivalent results were obtained when cells were transfected with 100 nM siRNA). Cells were treated with UC EVs (2.5 μg/ml), α_2_M that was activated for binding to LRP1 by reaction with methylamine (10 nM), S-PrP (40 nM), or vehicle, for 30 min as indicated. Phospho-ERK1/2 and total ERK1/2 were determined by immunoblot analysis. ERK1/2, extracellular signal–regulated kinase 1/2; EV, extracellular vesicle; LRP1, lipoprotein receptor–related protein-1; NMDA-R, *N*-methyl-d-aspartate receptor; NTC, nontargeting control; PrP^C^, cellular prion protein; S-PrP, recombinant soluble cellular prion protein; UC, ultracenrifugation.

To confirm the role of the NMDA-R and LRP1 in ERK1/2 activation by UC EVs, we silenced expression of *Lrp1* and *Grin1* with siRNA in PC-12 cells. *Grin1* encodes the essential GluN1 subunit in the NMDA-R. We also silenced expression of *Prnp*, which encodes PrP^C^ in PC-12 cells. Control cells were transfected with nontargeting control (NTC) siRNA. Figure 4*A* shows that the siRNAs specifically silenced the targeted genes without altering expression of nontargeted genes. Figure 4*B* shows that UC EVs activated ERK1/2 in cells transfected with NTC siRNA, as did the control NMDA-R–LRP1 ligands, S-PrP (40 nM) and purified α_2_M (10 nM), which was converted into the LRP1-recognized form by reaction with methylamine (42). In cells in which *Lrp1* or *Grin1* was silenced, the response to UC EVs was blocked, as was the response to S-PrP and α_2_M (Fig. 4, *C* and *D*), confirming that the NMDA-R–LRP1 system mediates ERK1/2 activation in PC-12 cells treated with UC EVs.

Mattei *et al.* (48) reported that tPA-initiated cell signaling requires target cell PrP^C^. However, we demonstrated that in PC-12 cells, PrP^C^ is not required for activation of cell signaling by S-PrP (21). Figure 4*E* confirms that when *Prnp* is silenced in PC-12 cells, ERK1/2 activation by S-PrP is not affected. Similarly, activation of ERK1/2 by UC EVs was not affected by *Prnp* gene silencing. By contrast, purified α_2_M failed to activate ERK1/2 when *Prnp* was silenced in PC-12 cells, suggesting a requirement for target cell PrP^C^ as an NMDA-R–LRP1 coreceptor for α_2_M, similar to that demonstrated with tPA previously (48). We conclude that UC EVs activate cell signaling in PC-12 cells by a mechanism that requires target cell NMDA-R and LRP1 but does not require target cell PrP^C^.

### EV-associated PrP^C^ is required for plasma EV–induced ERK1/2 activation and neurite outgrowth in PC-12 cells

The PrP^C^-specific monoclonal antibodies, POM1, POM2, POM3, and POM19, bind to defined epitopes in the structure of PrP^C^ (49). POM2, which binds to the tandem octarepeats in the N-terminal unstructured region of PrP^C^, is the only PrP^C^-specific antibody in this series that blocks signal transduction initiated by S-PrP (21).

**Figure 5:**
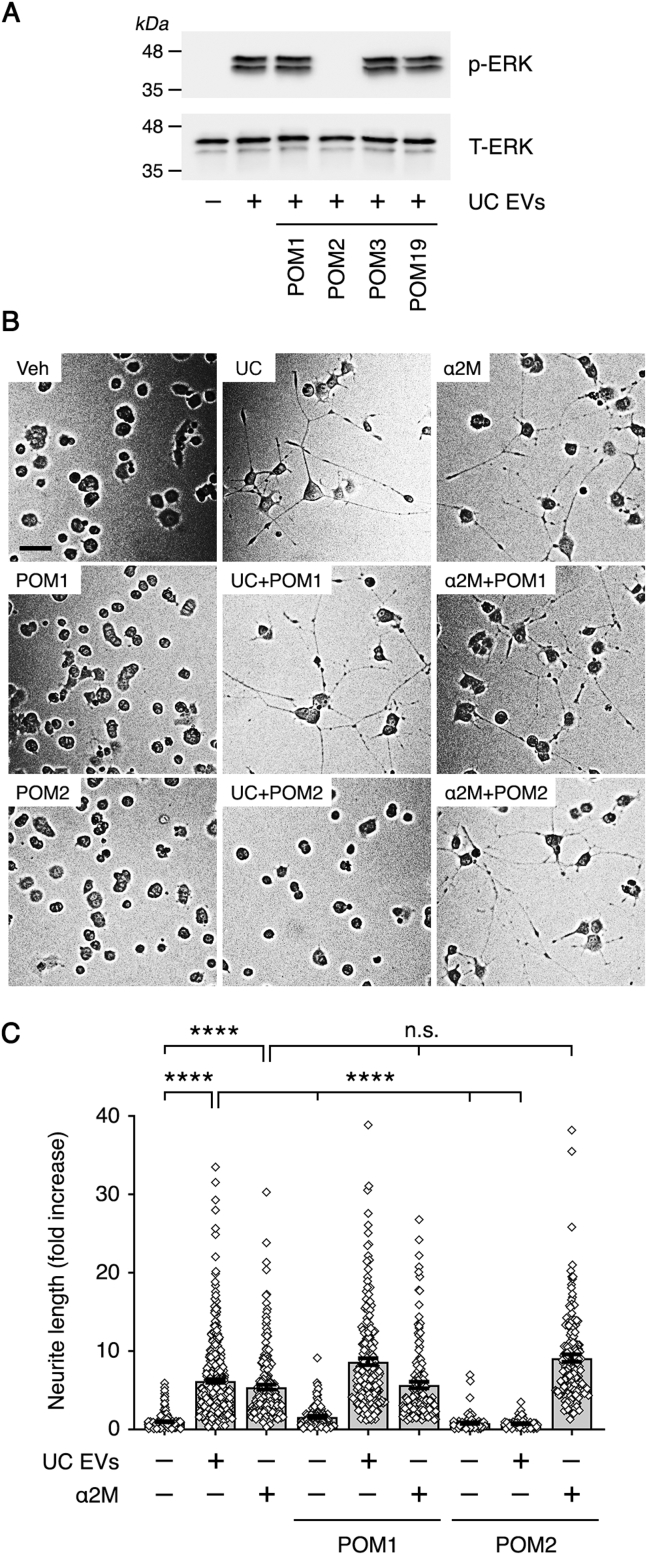
**PrP**^**C**^**-specific monoclonal antibody blocks the effects of human plasma EVs on PC-12 cell ERK1/2 activation and neurite outgrowth.***A*, PC-12 cells were treated with UC EVs (2.5 μg/ml) in the presence and absence of the PrP^C^-specific monoclonal antibodies: POM1, POM2, POM3, and POM19 (each at 10 μg/ml) for 30 min. Immunoblot analysis was performed to detect phospho-ERK1/2 and total ERK1/2. *B*, PC-12 cells were treated with UC EVs (2.5 μg/ml) or α_2_M that was activated for binding to LRP1 (10 nM) in the presence and absence of POM1 or POM2 (10 μg/ml), as indicated, for 48 h. Neurite outgrowth was examined. Representative images of cells are shown (the scale bar represents 50 μm). *C*, neurite outgrowth was quantified. For each replicate, neurites in 100 randomly selected cells were measured (data are expressed as “fold increase” compared with untreated control cells; mean ± SEM; n = 3; ∗∗∗∗*p* < 0.0001; ns, not significant). The datasets used to generate the first bar to the *left* (untreated control PC-12 cells) and the second bar (PC-12 cells treated with UC EVs) were identical to those shown in Figure 3. α_2_M, α_2_-macroglobulin; ERK1/2, extracellular signal–regulated kinase 1/2; EV, extracellular vesicle; LRP1, lipoprotein receptor–related protein-1; PrP^C^, cellular prion protein; UC, ultracentrifugation.

Figure 5*A* shows that POM2 (10 μg/ml) completely blocked ERK1/2 activation by UC EVs (2.5 μg/ml). POM1, POM3, and POM19 (10 μg/ml) were without effect. POM2 also blocked the ability of UC EVs to promote neurite outgrowth in PC-12 cells, as shown in the representative images in Figure 5*B* and in summary form in Figure 5*C*. POM-1 was ineffective.

In control experiments, methylamine-activated α_2_M promoted neurite outgrowth in PC-12 cells, an anticipated consequence of its known interaction with the NMDA-R–LRP1 receptor system (41, 42, 43). However, the activity of α_2_M was not inhibited by POM1 or POM2, despite the apparent requirement for PrP^C^ as an NMDA-R–LRP1 coreceptor for α_2_M, identified in *Prnp* gene-silencing studies (Fig. 4*E*). These results suggest that, in experiments with plasma EVs, POM2 targets EV-associated PrP^C^ and not PC-12 cell PrP^C^.

**Figure 6:**
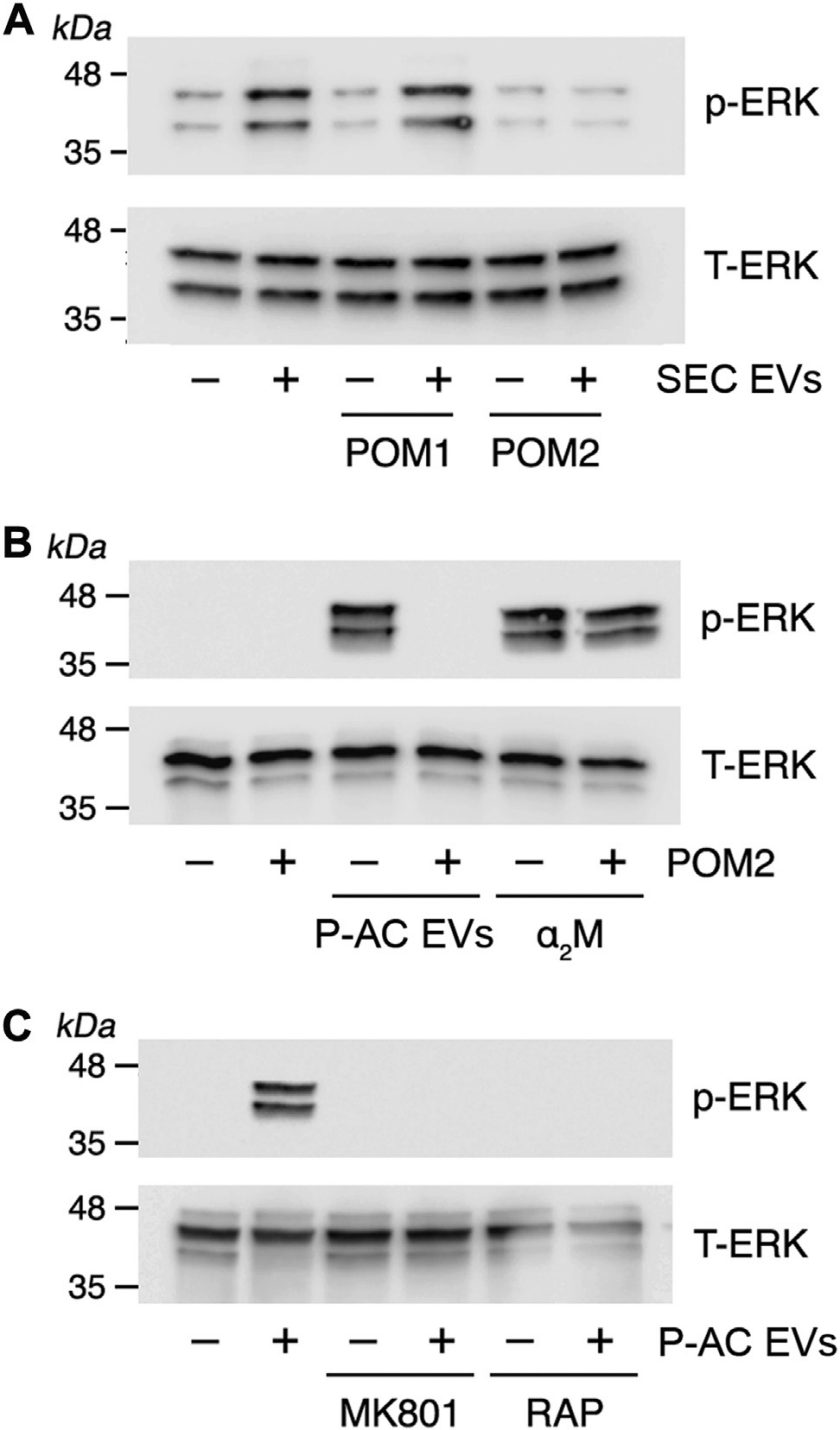
**Highly purified human plasma EVs activate ERK1/2 by an NMDA-R–LRP1-dependent pathway.***A*, PC-12 cells were treated with SEC EVs (2.5 μg/ml) in the presence and absence of POM1 or POM2 (10 μg/ml) for 30 min as indicated. Phospho-ERK1/2 and total ERK1/2 were determined by immunoblot analysis. *B*, PC-12 cells were treated with P-AC EVs (2.5 μg/ml) or α_2_M that was activated for binding to LRP1 (10 nM), in the presence and absence of POM2 (10 μg/ml) for 30 min as indicated. Phospho-ERK1/2 and total ERK1/2 were determined by immunoblot analysis. *C*, PC-12 cells were pretreated with MK801 (1.0 μM) or with RAP (150 nM) for 30 min, as indicated, and then with P-AC EVs (2.5 μg/ml) for 30 min. Phospho-ERK1/2 and total ERK1/2 were determined by immunoblot analysis. α_2_M, α_2_-macroglobulin; ERK1/2, extracellular signal–regulated kinase 1/2; EV, extracellular vesicle; LRP1, lipoprotein receptor–related protein-1; NMDA-R, *N*-methyl-d-aspartate receptor; P-AC, phosphatidylserine affinity chromatography; RAP, receptor-associated protein; SEC, size-exclusion chromatography.

To confirm that EV PrP^C^ is responsible for the effects of human plasma EVs on PC-12 cells, we examined more highly purified plasma EV preparations. Figure 6*A* shows that SEC EVs activated ERK1/2, and the response was blocked by POM2 but not POM1. P-AC EVs, which were depleted of α_2_M, also activated ERK1/2, and the response was blocked by POM2 (Fig. 6*B*). In control experiments, methylamine-activated α_2_M activated ERK1/2 as anticipated; however, as was the case in the neurite outgrowth studies, α_2_M-induced ERK1/2 activation was not inhibited by POM2.

Figure 6*C* shows that MK801 and RAP inhibited ERK1/2 activation in PC-12 cells treated with P-AC EVs, confirming an essential role for the NMDA-R–LRP1 receptor system with this highly purified EV preparation.

### Astrocyte EVs activate ERK1/2 in PC-12 cells by a PrP^C^-dependent pathway

EVs regulate cell physiology by autocrine, paracrine, and endocrine pathways (1, 2, 3, 4). To model a paracrine interaction that may occur in the nervous system, we harvested EVs from cultured rat astrocytes and examined their ability to trigger signal transduction in PC-12 cells, which are neuron-like cells.

**Figure 7:**
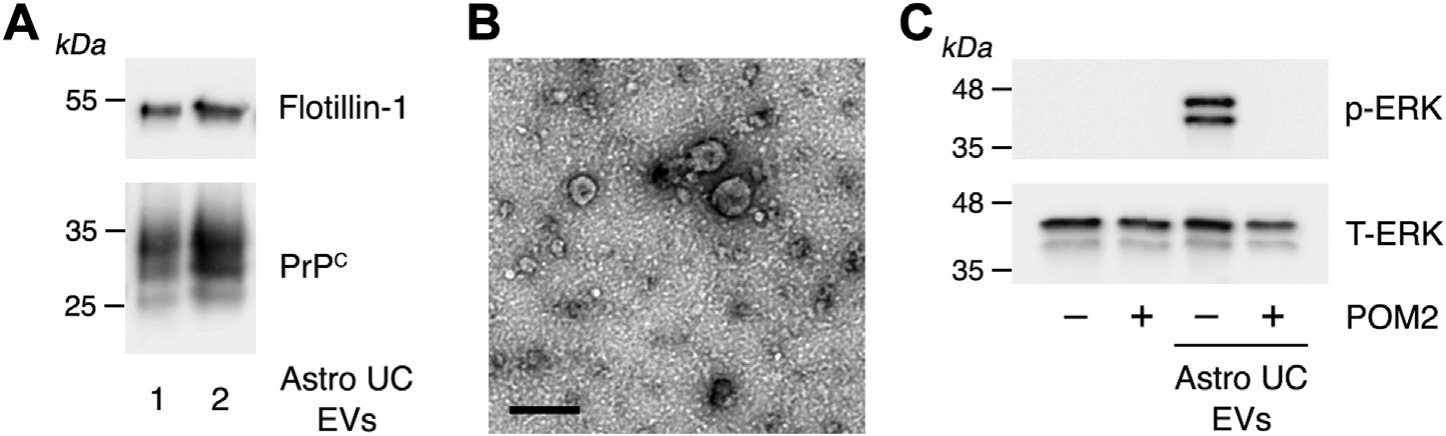
**EVs harvested from astrocyte cultures activate ERK1/2 in PC-12 cells by a POM2-dependent mechanism.***A*, two separate preparations of EVs harvested from cultured astrocytes by UC were subjected to immunoblot analysis to detect flotillin-1 and PrP^C^. *B*, astrocyte EVs were examined by TEM following negative staining with uranyl acetate (the scale bar represents 200 nm). *C*, PC-12 cells were treated with EVs harvested from astrocyte cultures (astro UC EVs, 2.5 μg/ml), in the presence and absence of POM2 (10 μg/ml) for 30 min, as indicated. Phospho-ERK1/2 and total ERK1/2 were determined by immunoblot analysis. ERK1/2, extracellular signal–regulated kinase 1/2; EV, extracellular vesicle; PrP^C^, cellular prion protein; TEM, transmission electron microscopy; UC, ultracentrifugation.

Figure 7*A* shows that the EV biomarker, flotillin-1, is present in EVs harvested from astrocytes. PrP^C^ also was present in astrocyte EVs, as determined by IB analysis. Representative TEM images of astrocyte EVs are shown in Figure 7*B*. Astrocyte EVs (2.5 μg/ml) activated ERK 1/2 in PC-12 cells (Fig. 7*C*). The response was blocked by POM2.

## Discussion

In this study, we demonstrated that EVs, isolated from human plasma, activate cell signaling and promote neurite outgrowth in PC-12 cells. Although the ability of EVs to promote neurite outgrowth is previously reported (28, 29, 30, 31, 32), we describe a novel mechanism underlying this response in which EV-associated PrP^C^ engages target cell NMDA-R–LRP1 complex. Because plasma EV preparations, prepared by UC alone, included protein contaminants such as α_2_M, which promotes PC-12 cell neurite outgrowth when converted into the LRP1-recognized conformation (41, 42, 43), we implemented purification methods in addition to UC to further enrich plasma EV preparations. The activities of UC EVs were replicated with SEC EVs and P-AC EVs, in which α_2_M was present at very low or undetectable levels.

The activities demonstrated here for human plasma EVs, which contained PrP^C^, replicated those observed previously with S-PrP (21). Plasma EV activity was entirely inhibited by the PrP^C^-specific antibody, POM2, but not by POM1, POM3, or POM19. As previously demonstrated with S-PrP (21), the effects of human plasma EVs on PC-12 cell signaling and neurite outgrowth were inhibited by antagonists of the NMDA-R (MK801) and LRP1 (RAP). Silencing expression of *Lrp1* or *Grin1* also blocked human plasma EV activity. Taken together, these results indicate that the PC-12 cell NMDA-R–LRP1 receptor complex recognizes S-PrP and EV-associated PrP^C^ similarly.

We performed a number of experiments to confirm that POM2 disrupts the response of PC-12 cells to EVs by targeting EV-associated PrP^C^ and not PC-12 cell PrP^C^. First, we silenced *Prnp* in PC-12 cells and showed that cell signaling in response to human plasma EVs was not inhibited. Although *Prnp* gene silencing in PC-12 cells inhibited the response to purified methylamine-activated α_2_M, POM2 had no effect on the activity of α_2_M, indicating that POM2 does not target PC-12 cells even in cases in which target cell PrP^C^ may be required as an NMDA-R–LRP1 coreceptor. Our results with POM2 were supported by studies with SEC and P-AC EVs, which contained substantially decreased levels of plasma protein contaminants.

Based on results reported here and previously (21, 48, 50, 51), we hypothesize that PrP^C^ exists in at least three distinct states that interact with the NMDA-R–LRP1 receptor complex. First, PrP^C^ in neuronal plasma membranes laterally associates with LRP1 in the same cell, and this interaction controls PrP^C^ trafficking, including PrP^C^ translocation to the cell surface after biosynthesis and endocytosis (50, 51). When PrP^C^ undergoes endocytosis, it transfers from lipid rafts to clathrin-coated pits in a process promoted by copper (52). LRP1 transiently associates with lipid rafts but then translocates to clathrin-coated pits as well (53). It is reasonable to speculate that lipid raft–associated proteins, such as PrP^C^, which associate with LRP1, may shuttle together with LRP1 between plasma membrane microdomains.

Soluble PrP^C^ derivatives, released by a disintegrin and metalloproteinase domain–containing protein proteases, constitute a second state of PrP^C^ that may interact with the NMDA-R–LRP1 receptor system (21). These soluble PrP^C^ derivatives trigger cell signaling, promote neurite outgrowth, and enhance neuronal differentiation (21, 54, 55). EV-associated PrP^C^ represents a third state of PrP^C^ that engages the NMDA-R–LRP1 receptor assembly. Although there is substantial evidence that the interaction of PrP^C^ with the NMDA-R–LRP1 receptor complex is mediated by direct association of PrP^C^ with LRP1 (48, 50, 51), membrane-anchored PrP^C^ also is reported to interact with the NMDA-R, at least functionally, in neurons (56). By binding copper and facilitating oxidation of nitric oxide to NO^+^, PrP^C^ promotes post-translational modification of the NMDA-R in synapses. This reaction is reported to be neuroprotective (56).

Most of our studies were performed with EVs harvested from human plasma; however, we also performed experiments with EVs isolated from cultured astrocytes. These EVs carried PrP^C^ and replicated the effects of plasma EVs on PC12 cell signaling, activating ERK1/2 *via* a POM2-inhibited pathway. PrP^C^ was previously identified in astrocyte EVs and shown to facilitate movement of these EVs across neuronal surfaces (57). This interaction may be critical for ultimate delivery of the EVs to target cells in the central nervous system (57).

The interaction of EV-associated PrP^C^ with the NMDA-R–LRP1 receptor complex in target cells constitutes a mechanism by which EVs may mediate cell–cell communication independently of cargo transfer. In this pathway, PrP^C^-carrying EVs selectively activate cell signaling in cells that express the NMDA-R–LRP1 receptor complex. In the nervous system, multiple cell types may express NMDA-R–LRP1 complex, including but not limited to neurons, astrocytes, and Schwann cells (41, 58, 59). These cells represent candidate targets for PrP^C^-carrying EVs. Given the known heterogeneity in EVs, understanding the variability in PrP^C^ levels in EVs, produced by various cells, is an important future goal. The ability of cells in the nervous system to respond to PrP^C^-carrying EVs may be regulated not only by expression of LRP1 and the NMDA-R but also by shedding of the LRP1 from cell surfaces (60, 61).

Although we did not directly demonstrate binding of EV-associated PrP^C^ to target cell LRP1 or the NMDA-R, our cell signaling results may be extended to suggest that target cell NMDA-R–LRP1 complex forms a direct physical association with EVs through EV PrP^C^. We hypothesize that multiple copies of target cell LRP1 may engage distinct PrP^C^ monomers displayed by a single EV. This type of interaction would strengthen the target cell–EV interface and may facilitate membrane fusion so that cargo is transferred from the EV to the target cell. Fully elucidating the role of LRP1 in EV trafficking will be an important goal. A second goal will be to determine whether interaction of EVs with LRP1 contributes to the diverse biological activities of LRP1, identified in conditional gene deletion studies, in the nervous system, and other tissues (22, 23, 24, 62).

## Experimental procedures

### Proteins and reagents

EVs are largely retained when platelet-poor plasma is prepared by methods applied in transfusion medicine and blood banking (63). We obtained FFP from the UCSD Transfusion Medicine service and studied the FFP without patient identifiers. This work was approved by the UCSD Institutional Review Board for Human Investigation. FFP units were divided into sections without thawing. In this manner, individual samples from the same unit could be studied without more than one freeze–thaw cycle.

α_2_M was purified from human plasma as previously described (64). Final preparations were homogeneous, as determined by SDS-PAGE. The concentration of α_2_M was determined by the absorbance at 280 nm, applying an *A*^*1%*^_*1 cm*_ of 8.93 (65). The activity of α_2_M as a protease inhibitor was determined using the method of Ganrot (44). Trypsin and soybean trypsin inhibitor were from Sigma–Aldrich. Standard curves were generated using known amounts of purified α_2_M.

α_2_M was converted into the LRP1-recognized conformation by reaction with 200 mM methylamine HCl, pH 8.0, as previously described (42, 66). S-PrP was expressed, purified, and authenticated as described by us (21). Endotoxin-free monomeric RAP was provided by Dr Travis Stiles (Novoron Bioscience). NGF-β was purchased from Invitrogen. MK801 was from Cayman Chemical Company. PS-specific antibody (clone 1H6) was from EMD Millipore. The PrP^C^-specific monoclonal antibodies POM1, POM2, POM3, and POM19 are previously described (49).

### Cell culture

PC-12 cells were from the American Type Culture Collection (CRL-1721) and subjected to quality control tests by the American Type Culture Collection. PC-12 cells were cultured in Dulbecco's modified Eagle's medium (high glucose; Gibco) containing 10% heat-inactivated fetal bovine serum (FBS) (Gibco), 5% heat-inactivated horse serum (HyClone), penicillin (100 units/ml), and streptomycin (1 mg/ml) in plates coated with 10 μg/ml type IV collagen (Sigma–Aldrich). Cells were passaged no more than eight times.

Astrocytes were isolated from Sprague–Dawley rat pup brains, as previously described (67). In brief, cortices were dissected from the forebrain and surrounding meninges and then mechanically and enzymatically dissociated using the Neural Tissue Dissociation Kit P (Miltenyi Biotec). Mixed glial cultures were established in Dulbecco's modified Eagle's medium/F-12 medium supplemented with GlutaMAX (Gibco), 10% FBS, and 100 units/ml antibiotic–antimycotic (Gibco). After culturing for 10 to 14 days, microglia and oligodendrocytes were removed by shaking. The astrocytes were collected by trypsinization and replated at 3.5 × 10^5^ cells/well on poly-d-lysine-coated surfaces. Experiments were performed within 48 h of completing the isolation procedure.

### Gene silencing

Rat-specific ON-TARGETplus SMARTpool siRNA, targeting *Lrp1*, the GluN1 subunit of the NMDA-R (*Grin1*), membrane-anchored PrP^C^ (*Prnp*), and pooled NTC siRNA were from Horizon Discovery. PC-12 cells (2 × 10^6^) were transfected with siRNA by electroporation using the Cell Line Nucleofector Kit V (Lonza), following the manufacturer's instructions. Briefly, cell suspensions were combined with *Lrp1*-specific siRNA (300 nM), *Grin1*-specific siRNA (300 nM), *Prnp*-specific siRNA (100 nM), or NTC siRNA (100 or 300 nM in each study, to match the specific siRNA), and electroporated with the PC-12-specific program in a Nucleofector 2b device. siRNA concentrations were selected to achieve similar levels of gene silencing at the mRNA level and were within the concentration ranges recommended by Lonza. Gene silencing was determined 48 h after transfection by RT–quantitative PCR. Experiments were performed 48 h after transfection.

### Isolation of EVs by sequential UC

Human FFP was subjected to centrifugation at 5000*g* for 10 min at 4 °C to ensure removal of platelets and cellular debris. The supernatant was collected, and larger EVs were precipitated by UC for 2 h at 20,000*g* at 4 °C (Avanti J Ultracentrifuge; Beckman Coulter). The supernatant, which included smaller EVs, such as exosomes, was collected and subjected to UC at 100,000*g* for 18 h at 4 °C. The pellet was resuspended in sterile PBS (20 mM sodium phosphate, 150 mM NaCl, pH 7.4), washed by UC at 100,000*g* for 2 h at 4 °C (Opti-Max E, MLS-50 swinging-bucket rotor; Beckman Coulter), and resuspended again in sterile PBS for experiments or further purification.

To collect EVs from cultured astrocytes, cells were maintained in medium supplemented with EV-depleted FBS. The EVs were then collected over 18 h in serum-free medium (SFM) and isolated by sequential UC.

### SEC

EVs that were isolated by UC were subjected to molecular exclusion chromatography on a 40 × 1.0 cm Sepharose CL-6B column. The flow rate was adjusted to 100 μl/min, and serial 750 μl fractions were collected. The absorbance at 280 nm was determined for each fraction. Protein content in each fraction was determined by bicinchoninic acid assay, and immunoblotting was performed to detect flotillin-1. Early eluting flotillin-1-positive fractions were pooled and referred to as SEC EVs.

### PS affinity chromatography

PS-specific antibody was coupled to cyanogen bromide–activated Sepharose CL-4B (GE Healthcare). The coupling ratio was 0.25 mg antibody per millliter of resin. UC EVs were diluted in PBS and cycled through the column for 4 h at 4 °C. The column was washed extensively with PBS until the absorbance at 280 nm was <0.005. The EVs were eluted in 1.0 ml fractions by pulse exposure to 100 mM glycine, pH 3.0, and immediately quenched with 1.5 M Tris–HCl, pH 8.0. The column was regenerated and effective for up to five P-AC EV purification procedures. P-AC EVs were re-established in PBS.

### NTA

EV suspensions were analyzed using a NanoSight NS300 instrument equipped with a 405 nm laser (Malvern). Vortexed samples were pushed through a fluidics flow chamber at a constant flow rate using a syringe pump at room temperature. Each sample was measured in triplicate with an acquisition time of 30 s and detection threshold setting of 3. Data were captured and analyzed with NTA software, version 2.3 (Malvern Panalytical).

### Immunoblot analysis

EVs were pelleted at 100,000*g* for 2.5 h at 4 °C and resuspended in 1% SDS with sonication at 37 °C for 5 min. Samples were boiled for 5 min in 2 × Laemmli sample buffer (Bio-Rad) containing 50 mM DTT, subjected to 4 to 15% SDS-PAGE, and electrotransferred to polyvinylidene fluoride membranes. The membranes were blocked with 5% nonfat dried milk and incubated with primary antibodies (1:1000 dilution) that detect flotillin-1 (BD Biosciences), heat shock protein-70 (Cell Signaling Technology), tumor susceptibility gene 101 (Abcam), CD9 (Novus Biologicals), CD81 (Novus Biologicals), golgi matrix protein 130 (BD Biosciences), α_2_M (Abcam), fibrinogen γ chain (Invitrogen), and PrP^C^ (Abcam). The membranes were washed and incubated with horseradish peroxidase–conjugated secondary antibody (Jackson Laboratories). Immunoblots were developed using Radiance Q chemiluminescent substrate (Azure Biosystems) in the Azure c300 digital imager (Azure Biosystems).

To detect PrP^C^ in UC and SEC EV preparations, samples were immunoprecipitated using POM2 + POM19 coupled to Dynabeads Protein-G (Thermo Fisher Scientific). EV preparations were incubated with the beads, with end-over-end rotation, for 12 h. The beads were then extensively washed. Associated proteins were eluted in 2 × Laemmli sample buffer for IB analysis. To lessen the amount of antibody coeluting in the SDS, the Dynabeads and associated IgGs were pretreated with 5.0 mM bis(sulfosuccinimidyl)suberate.

### ERK1/2 phosphorylation in EV-treated PC-12 cells

PC-12 cells were transferred to SFM for 2 h and preincubated with 150 nM RAP or with 1.0 μM MK801 for 30 min, as indicated. The cells were then treated with EVs (2.5 μg/ml), 10 nM activated α_2_M, 40 nM S-PrP, or vehicle for 30 min. In some studies, POM1, POM2, POM3, or POM19 (10 μg/ml) was added together with the EVs or with α_2_M. The cells were rinsed twice with ice-cold PBS and extracted in radioimmunoprecipitation buffer (PBS with 1% Triton X-100, 0.5% sodium deoxycholate, 0.1% SDS, protease inhibitor mixture, and phosphatase inhibitor mixture). Equivalent amounts of cellular protein (20 μg), as determined by DC Protein Assay (Bio-Rad), were subjected to 10% SDS-PAGE and electrotransferred to polyvinylidene fluoride membranes. Immunoblotting was performed to detect phosphorylated ERK1/2 and total ERK1/2 (Cell Signaling Technologies; 1:1000 dilution).

### Neurite outgrowth

PC-12 cells were plated at 1 × 10^5^ cells/well and maintained in serum-containing medium for 24 h. The medium was then replaced with SFM supplemented with activated α_2_M (10 nM), RAP (150 nM), MK801 (1.0 μM), NGF-β (50 ng/ml), EVs (2.5 μg/ml), POM1 (10 μg/ml), POM2 (10 μg/ml), combinations of these reagents, or vehicle for 48 h, as indicated. At the end of each incubation, the cells were imaged by phase contrast microscopy, using a Leica DMi8 microscope (Leica Microsystems) equipped with a Leica DFC3000 G digital camera and Leica Application Suite X software. Neurite length was determined in 100 cells per replicate using ImageJ software (the National Institutes of Health) (n = 3/condition).

### TEM

Isolated EVs were incubated on formvar/carbon-coated 100-mesh copper grids for 10 min, washed with water, and stained with 2% uranyl acetate aqueous solution for 1 min. Grids were viewed using a JEOL 1200EX II transmission electron microscope and photographed using a Gatan digital camera.

### LC–MS/MS

UC EVs (n = 3) and P-AC EVs (n = 4) were reduced, alkylated, and digested with the Arg/Lys-specific protease, trypsin. Peptides were passed through C18 spin tips (Thermo Fisher Scientific) and eluted in 80% acetonitrile and 0.1% formic acid. Samples were then vacuum dried, equilibrated in 1% acetonitrile and 0.1% formic acid, packed into 70 μm C18 infused capillaries, and eluted in a positive ion nanospray with a 1 to 90% acetonitrile gradient using an Agilent 1200 series liquid chromatography injection system. Peptides were detected with an LTQ OrbiTrap XL mass spectrometer using Xcalibur 2.1 (Thermo Fisher Scientific). For assignment, raw files were searched against the *Homo sapiens* proteome (UniProt Taxonomy ID: 9606, release 2015_02), containing 20,610 entries, using Proteome Discoverer Software 2.0 with SEQUEST HT and MS Amanda search engines (Thermo Fisher Scientific). Our search parameters identified fixed modifications, including cysteine carbamidomethylation, variable methionine oxidation, lysine carbamylation, and N-terminal acetylation and oxidation. The maximum number of missed cleavages permitted was two. Mass tolerance for precursor ions was set to 50 ppm and for fragment ions, 0.6 Da. Peptides with an Xcorr threshold ≤1% were subjected to validation through the MS Amanda search engine. A strict peptide false positive rate of 5% was used to accept proteins based on spectral match. Each distinct EV sample was analyzed in technical duplicates. Identified peptides were categorized as cellular or extracellular. The former were assigned to EVs and the latter to plasma proteins that may have been associated with EV surfaces or contaminants. The percent of protein attributed to EVs *versus* plasma proteins in each EV preparation was determined by spectral counts.

### Statistical analysis

Statistical analysis was performed using GraphPad Prism 9 (GraphPad Software, Inc). Results are presented as the mean ± SEM. Comparisons between two groups were performed using two-tailed unpaired *t* tests. When more than two groups were compared, we performed one-way analysis of variance followed by Tukey's multiple comparisons test (∗*p* < 0.05, ∗∗*p* < 0.01, ∗∗∗*p* < 0.001, and ∗∗∗∗*p* < 0.0001).

## Data availability

The raw LC–MS/MS files for the characterization of EVs are available in the public repository Figshare (10.6084/m9.figshare.14720970).

## Supporting information

This article contains supporting information .

## Conflict of interest

The authors declare that they have no conflicts of interest with the contents of this article.
