# Supplementary material for: Cellular prion protein in human plasma–derived extracellular vesicles promotes neurite outgrowth via the NMDA receptor–LRP1 receptor system
Source: J Biol Chem. 2022 Jan 25;298(3):101642. doi: 10.1016/j.jbc.2022.101642 (PMC8861162; doi:10.1016/j.jbc.2022.101642)
Supplement: Supplemental Figure S1 [file mmc1.pdf]

Cellular prion protein in human plasma-derived extracellular vesicles promotes neurite outgrowth via the NMDA receptor-LRP1 receptor system

Steven L. Gonias, Michael A. Banki, Pardis Azmoon, Haylie K. Romero, Christina J. Sigurdson, Elisabetta Mantuano, and Wendy M. Campana

Supporting information

Supplemental Figure 1 – Uncropped immunoblot for PrP^C in human plasma UC EVs performed after IP/IB. The blot on the left was probed first with PrP^C-specific antibody and then with horseradish peroxidase-conjugated secondary antibody. As a control, the identical sample was probed only with secondary antibody, showing that the major band at 55-kDa was non-specific.

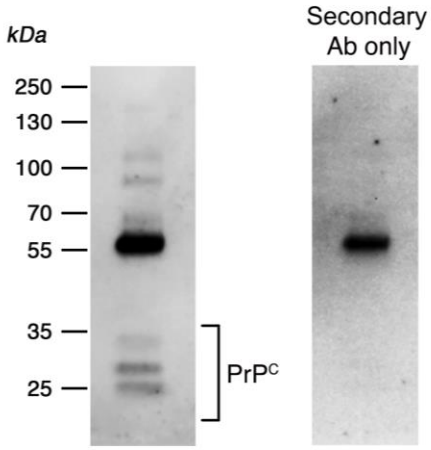

Supplemental File 1 – Complete list of identified peptides in our LC-MS/MS analysis of UC EVs and P-AC EVs (provided as separate Excel file)
